# Population subdivision of hydrothermal vent polychaete *Alvinella pompejana* across equatorial and Easter Microplate boundaries

**DOI:** 10.1186/s12862-016-0807-9

**Published:** 2016-10-28

**Authors:** Sook-Jin Jang, Eunji Park, Won-Kyung Lee, Shannon B. Johnson, Robert C. Vrijenhoek, Yong-Jin Won

**Affiliations:** 1 Interdisciplinary Program of EcoCreative, The Graduate School, Ewha Womans University, Seoul, South Korea; 2 Division of Ecoscience, Ewha Womans University, Seoul, South Korea; 3 Monterey Bay Aquarium Research Institute, Moss Landing, CA 95039-9644 USA

**Keywords:** Hydrothermal vent, Polychaeta, Metapopulations, Divergence, Gene flow

## Abstract

**Background:**

The Equator and Easter Microplate regions of the eastern Pacific Ocean exhibit geomorphological and hydrological features that create barriers to dispersal for a number of animals associated with deep-sea hydrothermal vent habitats. This study examined effects of these boundaries on geographical subdivision of the vent polychaete *Alvinella pompejana*. DNA sequences from one mitochondrial and eleven nuclear genes were examined in samples collected from ten vent localities that comprise the species’ known range from 23°N latitude on the East Pacific Rise to 38°S latitude on the Pacific Antarctic Ridge.

**Results:**

Multi-locus genotypes inferred from these sequences clustered the individual worms into three metapopulation segments — the northern East Pacific Rise (NEPR), southern East Pacific Rise (SEPR), and northeastern Pacific Antarctic Ridge (PAR) — separated by the Equator and Easter Microplate boundaries. Genetic diversity estimators were negatively correlated with tectonic spreading rates. Application of the isolation-with-migration (IMa2) model provided information about divergence times and demographic parameters. The PAR and NEPR metapopulation segments were estimated to have split roughly 4.20 million years ago (Mya) (2.42–33.42 Mya, 95 % highest posterior density, (HPD)), followed by splitting of the SEPR and NEPR segments about 0.79 Mya (0.07–6.67 Mya, 95 % HPD). Estimates of gene flow between the neighboring regions were mostly low (2 *Nm* < 1). Estimates of effective population size decreased with southern latitudes: NEPR > SEPR > PAR.

**Conclusions:**

Highly effective dispersal capabilities allow *A. pompejana* to overcome the temporal instability and intermittent distribution of active hydrothermal vents in the eastern Pacific Ocean. Consequently, the species exhibits very high levels of genetic diversity compared with many co-distributed vent annelids and mollusks. Nonetheless, its levels of genetic diversity in partially isolated populations are inversely correlated with tectonic spreading rates. As for many other vent taxa, this pioneering colonizer is similarly affected by local rates of habitat turnover and by major dispersal filters associated with the Equator and the Easter Microplate region.

**Electronic supplementary material:**

The online version of this article (doi:10.1186/s12862-016-0807-9) contains supplementary material, which is available to authorized users.

## Background

**Fig. 1:**
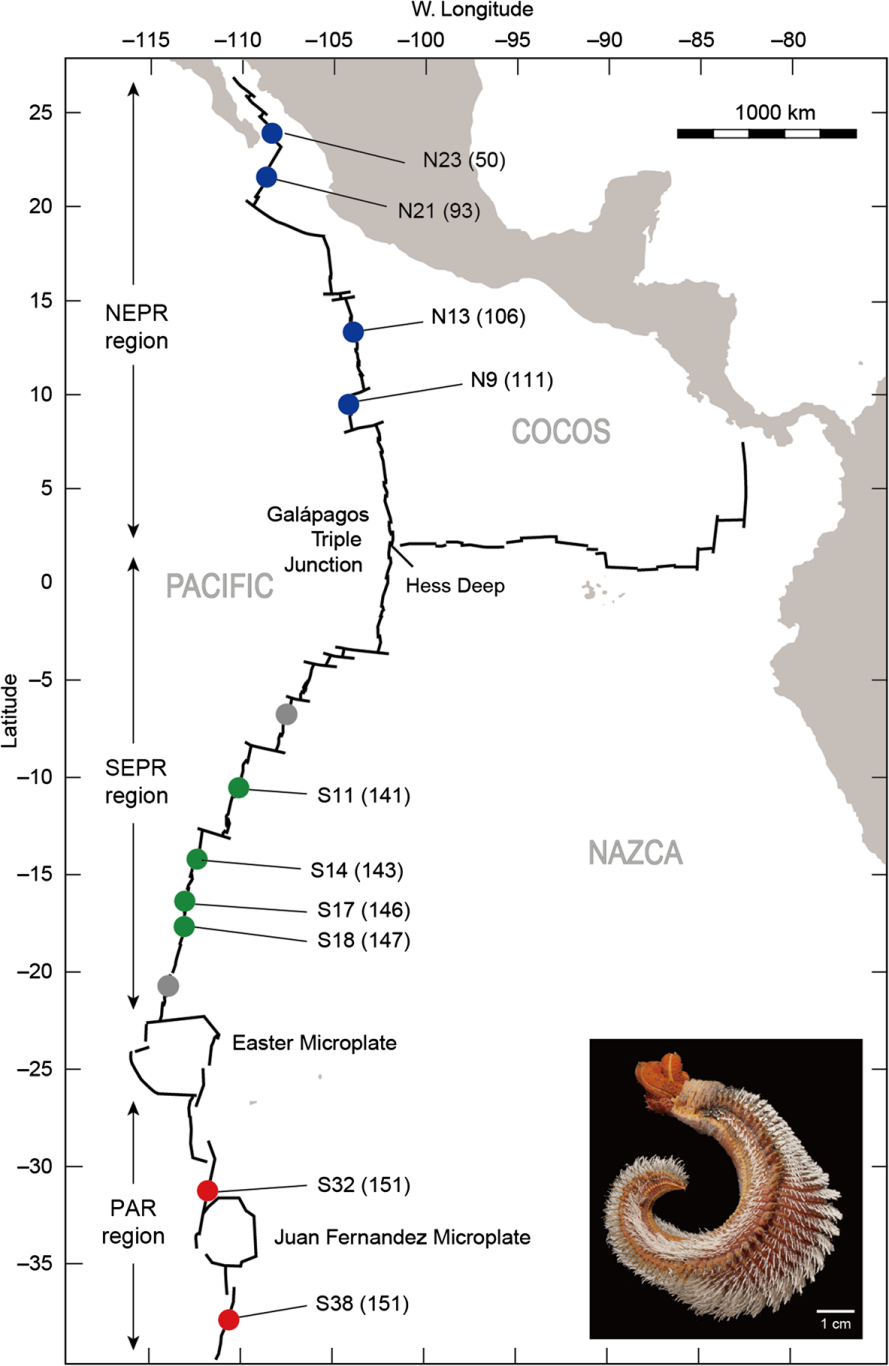
Map of the eastern Pacific ridges and *Alvinella pompejana* sampling locations along the ridge axes. Colored dots indicate sampled vent field locations: blue, green and red dots, sampling sites within the NEPR, SEPR and PAR regions. Gray dots represent records for *A. pompejana* but were not included in this study. The numbers in parenthesis following the locality names indicate tectonic spreading rate (mm/year). Inset photo of *A. pompejana* was supplied courtesy of G. W. Rouse, Scripps Institution of Oceanography, UCSD. Photo was taken from sample collections of the Alarcón Rise

The past 25 years of population genetic studies have revealed a number of physical and biological processes that shape the geographical structure, interpopulation connectivity and genetic diversity of deep-sea hydrothermal vent species (reviewed in [1]). Extrinsic factors, such as the geomorphology of oceanic ridges, deep oceanic currents and the temporal stability of vents, influence the genetic structure of vent species, and intrinsic factors, such as taxon-specific differences in larval development, larval duration, motility and behavior, affect connectivity [1–7]. The most intensively studied invertebrate animals, in these regards, inhabit the southeastern Pacific ridge systems (Fig. 1), composed of the northern and southern East Pacific Rise (NEPR and SEPR), the Galápagos Rift (GAR) and the northeastern Pacific Antarctic Ridge (PAR). Three metapopulation patterns have emerged from past studies (see Figure 4 in [1]); (1) the metapopulations typically exhibit geographical subdivision involving one or two partitions along the contiguous ridge axes, with relatively low genetic differentiation within metapopulation segments; (2) geographical boundaries of the partitions can vary among the co-distributed species; and (3) the taxon-specific effects of these boundaries as dispersal filters can range from complete isolation and speciation (vicariance) to limited (or no) dispersal impedance. The most consistent boundaries identified to date coincide with the Equatorial region, separating the NEPR + GAR axes from the SEPR axis, and the Easter Microplate region, separating the SEPR and PAR axes.

This study examined geographical population structure and connectivity of the Pompeii worm, *Alvinella pompejana* Desbruyères and Laubier, (Fig. 1) [8]. With a known distribution spanning 8300 km, this annelid is among the pioneer-species that settle first on newly formed hydrothermal chimneys on the southeastern Pacific ridge systems [9]. Its extraordinary thermal tolerance has attracted the attention of vent researchers for the past two decades [10–15]. The worms are covered by a “dense fleece” of epsilon proteobacterial episymbionts that contribute to their nutrition and thermal protection [16, 17]. Production of large (~200 μm) lecithotrophic larvae that arrest development in cold abyssal waters allows the worms to disperse great distances and be among the first to colonize nascent hot vents [9]. Its life history and behavioral traits appear to be optimized for exploiting the patchily distributed and highly ephemeral eastern Pacific hydrothermal vents [1]. Nonetheless, we have only rudimentary knowledge about the effects of these traits on the geographical structure and genetic connectivity of *A. pompejana* metapopulations.

Previous studies of *A. pompejana* identified distinct metapopulation segments separated by the Equatorial boundary. An examination of mitochondrial cytochrome-*c*-oxidase subunit-I (*mtCOI*) sequences (710 bp) in samples that ranged between 21°N to 32°S latitude identified distinct NEPR and SEPR metapopulation segments, but found no evidence for a distinct segment occupying the PAR axis [18]. A subsequent examination of *mtCOI*, multi-locus allozymes and four nuclear genes in *A. pompejana* samples ranging between 13°N and 21°S confirmed the distinct NEPR and SEPR segments [19, 20]. To date, however, multi-locus genetic markers have not been examined in samples from the extended range of *A. pompejana*, 23°N on the EPR to 38°S on the PAR, reported for the first time in this study.

Comprehensive geographical sampling and the application of multi-locus genetic markers have often improved or even contradicted previous inferences about genetic structure and the demographic history of vent species. For example, a multi-locus investigation revealed that SEPR populations of the siboglinid polychaete *Tevnia jerichonana* exhibited a broad zone of intergradation between distinct metapopulation segments occupying the NEPR and PAR axes [21]. Inferences based on *mtCOI* evidence alone reached different conclusions [4, 18]. Coykendall et al.’s [22] multi-locus study of the siboglinid polychaete *Riftia pachyptila* did not corroborate an earlier *mtCOI* study that concluded SEPR and PAR populations were partially isolated across the Easter Microplate boundary [18]. Multi-locus data also revealed a hybrid zone at the Easter Microplate boundary [23, 24] that was not recognized in more limited samples of the vent mussels *Bathymodiolus thermophilus* and *B. antarcticus* [2].

Computer simulations revealed that inferences about population subdivision and isolation-by-distance are limited by the number of independent gene loci examined and the completeness of population sampling [4]. Sampling gaps can create false evidence for subdivision and examinations of mitochondrial DNA alone occasionally provide signals for population subdivision that are discordant with evidence provided by independent nuclear genes (e.g., [25, 26]). To assess potential dispersal barriers associated with the Equatorial and Easter Microplate boundaries, we examined DNA sequences from *mtCOI* and 11 nuclear genes in geographical samples that extend the known range for *A. pompejana* northward to 23°N latitude in the Alarcón Basin and southward to 38°S on the Pacific Antarctic Ridge. The present study reports the most comprehensive geographical sampling and genetic analysis of this species, to date.

## Methods

### Samples

Samples were obtained during oceanic expeditions that spanned 21 years (Table 1, Fig. 1) with robotic manipulators or slurp guns on the human occupied vehicle (HOV) *Alvin* (Woods Hole Oceanographic Institution, WHOI) and the remotely operated vehicles (ROVs) *Tiburon* and *Doc Ricketts* (Monterey Bay Aquarium Research Institute, MBARI). Upon recovery at the surface, the samples were briefly stored in cold (2 °C) filtered seawater prior to dissection and tissue removal. Tissue samples were frozen at −70 °C on board the vessels and subsequently stored at −80 °C in the land based laboratories. DNA sequencing was conducted with subsamples of individuals from each of the sample localities. Genomic DNA was extracted from muscle tissue with the Qiagen Blood and Tissue kit, following manufacturer’s protocols (Qiagen, Hilden, Germany).

**Table 1:** Sampling localities

Location	°N Latitude	°E Longitude	Depth (m)	Dive^a^	Date	Sample size
23N	23.4	−108.6	2287	D752/D753	Apr-2015	9
21N	20.8	−109.1	2553/2542	T556/A3748	Apr-2003/Jan-2002	9
13N	12.8	−103.9	2623	A3036	Dec-1995	5
9N	9.8	−104.3	2506	A2849	Oct-1994	9
11S	−11.3	−110.5	2791	A3323	Dec-1998	9
14S	−14.0	−112.5	2619	A3324	Dec-1998	3
17S	−17.6	−113.2	2599	A3330	Jan-1999	5
18S	−18.4	−113.4	2629/2648/2654	A3331/A3332/A3333	Jan-1999	11
32S	−31.9	−112.0	2334/2333/2338	A3340/A3341/A3342	Jan-1999	10
38S	−37.8	−110.9	2222	A4089	Mar-2005	8

^a^ Dive numbers: (D….) ROV *Doc Ricketts*; (A….) HOV *Alvin*; and (T….) ROV *Tiburon*

### Molecular methods

Primer pairs were previously described for one mitochondrial and three nuclear protein-coding genes: *mtCOI*, cytochrome-*c-*oxidase sub-unit I [18]; *SAHH*, S-adenosylhomocysteine hydrolase; *GlobX*, globin *X*; and *PGM*, Phosphoglucomutase [20]. The *SAHH* marker includes part of an exon, whereas *GlobX* and *PGM* markers are composed entirely of introns.

We developed primer pairs for sequencing eight non-coding nuclear regions. High concentration DNA (100μg/μl) extracted from one individual from 14°S was sent to the National Instrumentation Center for Environmental Management (NICEM) at Seoul National University, Seoul Korea, for pyrosequencing on a Roche/454 Life Sciences Genome sequencer GS FLX machine. We obtained 28,222 reads with a total length of 9,804,791 base pairs. The pyrosequencing sequence reads were partitioned into protein-coding genes vs. non-coding DNA fragments identified through BLAST. Primer pairs were then designed for a subset of non-coding fragments containing > 450 base pairs (bp) and no poly (*n*) microsatellite repeats. Of these, we chose eight polymorphic loci, based on their PCR-efficacy, for further study: *AP_NC1*, *AP_NC3*, *AP_NC8*, *AP_NC20*, *AP_NC22*, *AP_NC28*, *AP_NC32* and *AP_NC43.* The primer pairs used for nested PCRs are provided (Additional file 1: Table S1).

PCRs for population screening of 12 markers were performed under the following conditions: the first PCR initial denaturation temperature at 94 °C/1 min followed by 35 cycles at 92 °C/30 sec, annealing at different temperatures depending on the markers from 45 to 58 °C/1 min, 72 °C/1 min with a single final extension at 72 °C/7 min, and second PCR repeated same conditions with first PCR, except for annealing temperature for 35 more cycles (Additional file 1: Table S1). All PCR products were sequenced with an ABI 3730xl automatic sequencer (Applied Biosystems, Foster City, CA).

### Statistical methods

We used PHASE v. 2.1.1 [27, 28] to resolve the phase of sequences with two or more heterozygous sites, and set the thresholds to 60 % with recombination model and stepwise mutation model. A number of sequences that could not be resolved in this manner needed to be cloned with three to five clones per individual. We used the pGEM-T easy vector (pGEM-T Easy Vector System, Promega, Madison, WI, USA) and DH5*α* (DH5*α* chemically competent *E. coli*, Enzynomics, Korea) for cloning and prepared the products for sequencing with the Hybrid-Q Plasmid rapidprep kit (GeneAll, Korea). All DNA sequences obtained in this study were deposited in GenBank (accession numbers: KX187433-KX189058, KX233878-KX233915 and KR868948-KR868986).

We used SITES [29] program to detect putatively non-recombinant segments within the DNA sequences from all eleven genetic markers for Isolation with Migration Analysis (IMa2), Tajima’s *D*, and Fu's *F*_S_ test. Putatively recombinant fragments were excluded and the longest remaining fragment was used for subsequent analyses (as previously recommended, [30]). We used Arlequin v. 3.5 [31] to estimate haplotype (*H*_*d*_) and nuclear (*π*) diversity indices, Tajima’s *D* [32] and Fu’s *F*_S_ [33]. Significance levels for Tajima’s *D* and Fu’s *F*s were corrected by Bonferroni method. Allelic richness and private allelic richness was estimated by rarefaction methods implemented in Hp-rare v. 1.0 [34]. Tests for linkage disequilibrium (LD) between markers were conducted with Genepop v. 4.2 [35]. A haplotype network for each marker was constructed with HapStar v. 0.7 [36] based on minimum spanning network resulting from Arlequin v. 3.5 [31].

### Analysis of population genetic structure

We used the PGDSpider [37] data format to prepare a table of multi-locus genotypes of nuclear genes for each individual. The individual genotypes were then examined with Structure v. 2.3.4 [38] to estimate the most probable number of discrete clusters (*K*) [38, 39]. We let *K* range from 1 to 10 and repeated the simulations at least five times for each value of *K* using admixture model with correlated allele frequencies among populations [40]. All simulations included 5 × 10^6^ MCMC (Markov chain Monte Carlo) generations after excluding the first 5 × 10^5^ as ‘burn-in’. The most probable value of *K* was estimated by the delta *K* method of Evanno et al. [41]. We used BayesAss v. 3 [42] to assess recent immigration events. Each run included 1 × 10^7^ iterations (−i) with random number seed (−s) and burn-in of 1 × 10^6^ (−b), a sample interval of 100 (−n), allele frequencies of 0.3 (−a), and inbreeding coefficients of 0.4 for (−f).

Hierarchical geographic subdivision was also tested with an analysis of molecular variance (AMOVA) [43]. Samples were grouped according to the results of Structure: northern East Pacific Rise (NEPR: 23N, 21N, 13N, and 9N); southern East Pacific Rise (SEPR: 11S, 14S, 17S, and 18S); and Pacific Antarctic Ridge (PAR: 32S and 38S) (Fig. 1). We used Arlequin v. 3.5 [31] with an option of locus by locus AMOVA based on multi-locus genotype data of nuclear genes and *mtCOI* sequence, respectively.

We used GenoDive v. 2.0b23 [44] to examine correlations (Mantel’s *r*) between pairwise genetic differentiation (*F*_ST_) based on nuclear genes and *mtCOI* gene separately, and geographic distances (from GeoDataSource <http://www.geodatasource.com/distance-calculator>). Because hierarchical subdivision can generate an apparent Isolation-by-Distance (IBD) pattern, we partitioned the samples into three regions, NEPR, SEPR, and PAR and applied a stratified Mantel test. The “Stratified” option in GenoDive randomly permutes the data within partitions to test for the residual correlations between the genetic and geographic matrices.

Finally, we examined correlations between degrees north latitude, tectonic spreading rates (mm/year) for the sampled localities, and average haplotype diversities (*H*_*d*_) for the twelve loci. Seafloor spreading rate was estimated under the NUVEL-1A model as implemented by the website < http://ofgs.aori.u-tokyo.ac.jp/~okino/platecalc_new.html>. Correlation analyses were implemented with SPSS v.21 (IBM Inc.).

### Isolation with migration analyses

We used the Isolation with Migration (IMa2) method [45, 46] to infer six demographic quantities of model parameters of population divergence from DNA sequence data. First, we applied the IMa2 model to analyze neighboring pairs of population clusters (NEPR vs. SEPR and SEPR vs. PAR) previously identified with the Structure analysis. Because IMa2 assumes no recombination, we used non-recombinant segments of each of the 11 nuclear loci resulting from the SITES [29] program with *mtCOI* sequence data. We applied the Hasegawa-Kishino-Yano (*HKY*) [47] mutation model for *mtCOI* (inheritance scalar, *H* = 0.25) and the Infinite Sites (*IS*) model [48] for the nuclear markers (*I* = 1.0). Each analysis included at least 1.0 × 10^7^ MCMC steps, and the first 1.0 × 10^5^ steps were discarded as burn-in, with 40 attempts of chain swapping per step, 40 chains with geometric heating, and *h1* and *h2* values of 0.975 and 0.75, respectively. The mutation scaled model parameters of IMa2 were transformed into corresponding demographic quantities (t as years, m as migration rate per generation, and *N* as effective population size) using a mutation rate of *mtCOI*. Due to the absence of confirmed mutation rates in *Alvinella pompejana*, we borrowed a substitution rate of 1.0–2.0 % per million years for *mtCOI*, as estimated for marine taxa partitioned across the Isthmus of Panama [49, 50]. Significance of migration rates between pairs of groups were analyzed with simple log-likelihood ratio tests [51].

The pairwise results were then used to analyze a three-population model (NEPR, SEPR and PAR) as described in [30, 46]. We used splitting times obtained from the previous analyses as prior information for the three-population model. Running conditions assumed the same mutation models for *mtCOI* and nuclear markers as in the 2-population analyses. After 5.0 × 10^5^ burn-in steps, at least 4.5 × 10^6^ MCMC steps proceeded with 100 chains with geometric heating, and *h1* and *h2* values of 0.99 and 0.75, respectively, for 200 attempts of chain swapping per step. We conducted this process multiple times with the same options using random seeds, and genealogies were saved every 100 Markov chain steps (default). Finally, we combined 2.0 × 10^5^ genealogies which were produced by multiple runs in L-mode, and tested significance with log-likelihood ratio test [52]. Demographic quantities of model parameters were visualized with the IMFig program [46].

## Results

### Genetic diversity

**Fig. 2:**
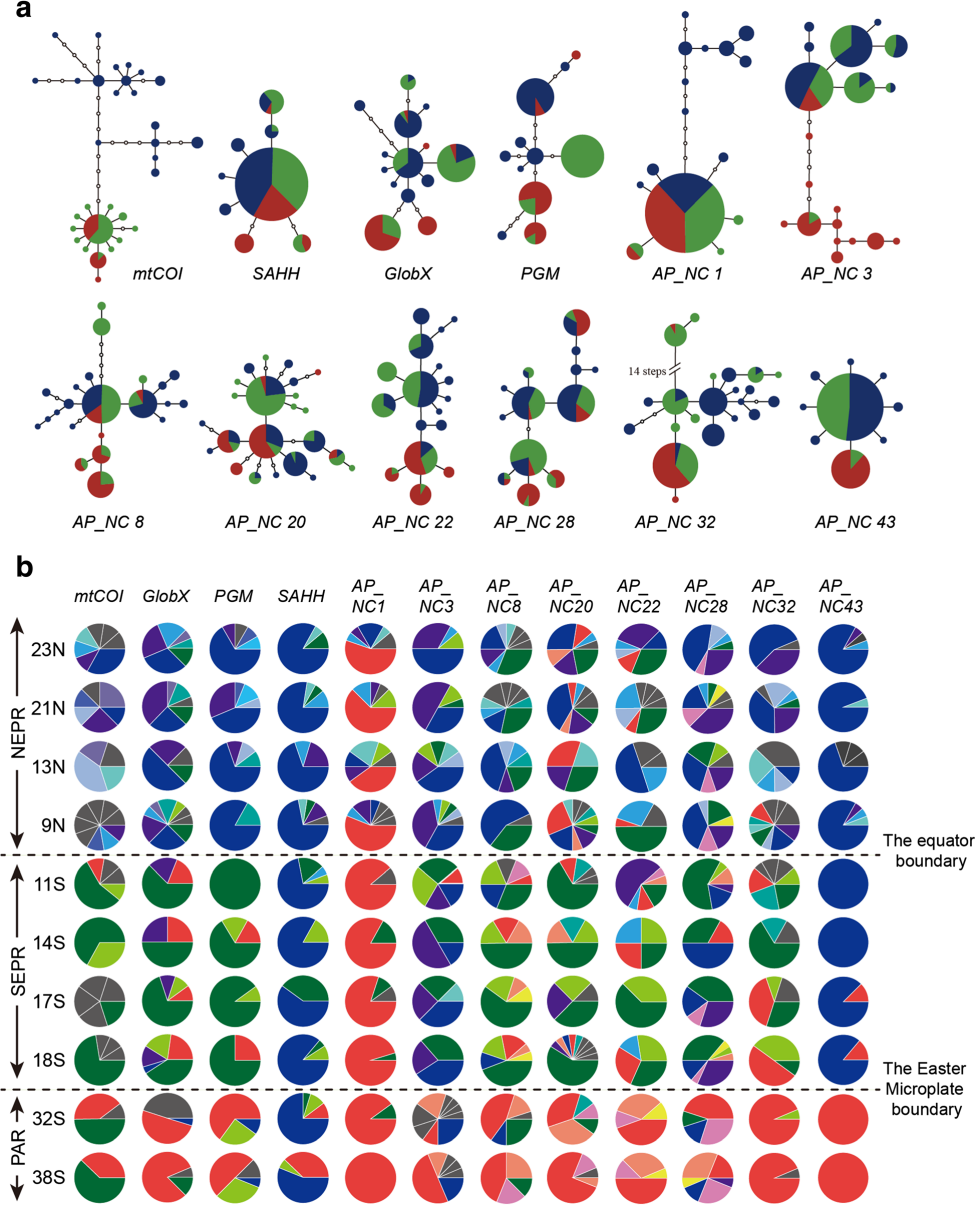
Haplotype networks of the twelve genetic loci. **a** The circles represent each unique haplotype which is scaled proportionally by relative frequency. The smallest white circles represent missed haplotypes in DNA samples and the line between the circles represents a single nucleotide difference between them. The three colors of the circles correspond to three geographic regions of the eastern Pacific ridges as illustrated in Fig. 1: *blue* = NEPR; *green* = SEPR; *red* = PAR. **b** Pie diagrams represent frequencies of twelve different loci in each sampling locality. For the sake of simplicity, some colors are omitted in the above haplotype network, but used here to represent different haplotypes. *Gray* color represents private alleles seen only at that single locality, and different private alleles are separated by a white line

The present suite of twelve genetic markers was polymorphic in nearly all of the vent samples (Table 2). Pairwise test for linkage disequilibrium among the eleven nuclear markers were non-significant for all the samples (*P*-value: 0.06–1.00). Noticeable regional differences existed in frequencies of the nuclear alleles and mitochondrial haplotypes. For example, the *mtCOI* haplotype network (Fig. 2a) exhibited haplotype clusters (shades of blue, green and red) that segregated between the NEPR (Fig. 2b, blue shades) and southern (SEPR + PAR) regions (Fig. 2b, red and green shades). Five mutational steps separated the NEPR and southern clusters. The predominant mitochondrial haplotypes in each region were complemented with numerous singletons (Fig. 2b, gray shades) and rare variants (i.e. *q*_i_ ≤ 0.05), but the NEPR variants tended to differ from predominant haplotypes by more mutations, leading to substantially greater nucleotide diversity in the NEPR samples (*π* for *mtCOI* in Table 2).

**Table 2:** Molecular diversity indices of the twelve loci examined across ten populations

Parameter	23N	21N	13N	9N	11S	14S	17S	18S	32S	38S	Total
Protein-coding loci											
*mtCOI* (*N*)^a^	9	8	5	9	9	3	5	11	10	8	77
*k*	19	14	10	18	4	1	4	4	2	1	39
*h*	7	6	4	9	5	2	5	4	3	2	31
*H*_*d*_	0.92	0.93	0.90	1.00	0.72	0.67	1.00	0.49	0.64	0.54	0.87
*π*	0.0138	0.0114	0.0078	0.0114	0.0017	0.0013	0.0031	0.0014	0.0015	0.0010	0.0099
*SAHH* (*N*)	18	18	10	18	18	6	10	22	20	16	156
*k*	3	3	2	5	4	2	2	4	5	3	8
*h*	9	4	3	5	4	2	2	3	4	3	8
*H*_*d*_	0.31	0.40	0.51	0.48	0.47	0.33	0.53	0.26	0.44	0.58	0.44
*π*	0.0013	0.0013	0.0014	0.0016	0.0022	0.0017	0.0027	0.0013	0.0023	0.0029	0.0020
*GlobX* (*N*)	16	16	8	16	16	4	10	22	20	16	144
*K*	8	8	3	10	4	4	7	7	6	4	16
*h*	6	6	4	9	3	3	4	5	3	3	14
*H*_*d*_	0.83	0.81	0.75	0.91	0.58	0.83	0.53	0.76	0.57	0.34	0.84
*π*	0.0045	0.0041	0.0029	0.0057	0.0041	0.0060	0.0046	0.0071	0.0063	0.0031	0.0069
*PGM* (*N*)	12	16	10	12	18	6	10	20	20	16	140
*k*	9	10	7	5	0	3	3	2	4	7	16
*h*	5	5	4	2	1	3	2	2	3	4	11
*H*_*d*_	0.58	0.73	0.53	0.30	0.00	0.60	0.20	0.39	0.53	0.68	0.79
*π*	0.0073	0.0079	0.0057	0.0046	0.0000	0.0043	0.0018	0.0024	0.0030	0.0065	0.0073
Non-coding loci											
*AP_NC1 *(*N*)	18	16	10	16	18	6	10	22	20	16	152
*k*	9	9	8	9	1	1	2	1	1	0	14
*h*	6	5	6	7	2	2	3	2	2	1	15
*H*_*d*_	0.68	0.61	0.84	0.69	0.21	0.33	0.38	0.09	0.19	0.00	0.41
*π*	0.0088	0.0082	0.0101	0.0090	0.0006	0.0009	0.0011	0.0002	0.0005	0.0000	0.0046
*AP_NC3 *(*N*)	18	18	10	18	18	6	8	22	20	16	154
*k*	3	3	6	6	8	2	3	2	9	11	17
*h*	4	4	6	7	5	3	4	3	9	6	19
*H*_*d*_	0.66	0.66	0.84	0.76	0.82	0.73	0.82	0.68	0.89	0.73	0.82
*π*	0.0022	0.0022	0.0043	0.0032	0.0064	0.0030	0.0032	0.0023	0.0106	0.0097	0.0077
*AP_NC8 *(*N*)	16	14	10	14	16	6	10	18	20	16	140
*k*	6	7	5	2	10	8	8	9	4	4	16
*h*	9	9	6	3	6	4	4	6	5	4	22
*H*_*d*_	0.88	0.91	0.84	0.58	0.85	0.80	0.64	0.77	0.74	0.74	0.84
*π*	0.0056	0.0061	0.0051	0.0018	0.0091	0.0087	0.0084	0.0089	0.0048	0.0038	0.0075
*AP_NC20 *(*N*)	18	18	10	16	18	6	8	22	20	16	152
*k*	6	9	4	10	5	7	5	8	7	7	17
*h*	7	8	4	10	5	4	4	10	5	4	23
*H*_*d*_	0.86	0.83	0.82	0.92	0.56	0.80	0.82	0.66	0.76	0.44	0.85
*π*	0.0056	0.0063	0.0046	0.0066	0.0036	0.0074	0.0068	0.0044	0.0046	0.0031	0.0056
*AP_NC22 *(*N*)	16	14	10	18	18	4	8	22	18	16	144
*k*	4	6	4	3	5	3	1	3	3	3	15
*h*	6	8	4	4	6	4	2	4	4	4	17
*H*_*d*_	0.82	0.89	0.73	0.68	0.68	1.00	0.54	0.77	0.71	0.68	0.87
*π*	0.0042	0.0045	0.0045	0.0032	0.0044	0.0047	0.0015	0.0038	0.0025	0.0023	0.0051
*AP_NC28 *(*N*)	18	16	10	16	18	6	10	22	20	16	152
*k*	5	5	7	5	4	4	5	6	6	7	8
*h*	6	8	6	6	5	3	4	7	4	6	13
*H*_*d*_	0.75	0.84	0.89	0.82	0.61	0.73	0.78	0.77	0.71	0.83	0.84
*π*	0.0035	0.0046	0.0059	0.0052	0.0038	0.0057	0.0048	0.0046	0.0072	0.0078	0.0059
*AP_NC32 *(*N*)	16	16	8	18	18	6	10	20	16	16	144
*k*	2	6	6	12	21	4	17	16	16	1	32
*h*	3	6	5	10	7	3	4	3	2	2	21
*H*_*d*_	0.58	0.80	0.86	0.93	0.88	0.60	0.78	0.61	0.13	0.13	0.84
*π*	0.0017	0.0034	0.0067	0.0079	0.0140	0.0053	0.0218	0.0223	0.0055	0.0003	0.0130
*AP_NC43 *(*N*)	18	18	10	18	18	6	8	22	16	14	148
*k*	3	1	3	3	0	0	1	1	0	0	8
*h*	4	2	4	4	1	1	2	2	1	1	9
*H*_*d*_	0.31	0.11	0.53	0.31	0.00	0.00	0.25	0.25	0.00	0.00	0.46
*π*	0.0009	0.0003	0.0017	0.0009	0.0000	0.0000	0.0007	0.0007	0.0000	0.0000	0.0014
Totals											
Haplotypes (*h*)	72	71	56	76	50	34	40	51	45	40	
Singletons (*S*)	10	14	8	17	5	1	6	7	6	6	
Mean *H*_*d*_	0.68	0.71	0.76	0.70	0.53	0.62	0.61	0.54	0.53	0.47	
Richness^b^	2.21	2.29	2.36	2.27	1.93	2.08	2.05	1.92	1.90	1.80	
Privates^c^	0.95	1.06	1.19	0.95	0.50	0.40	0.60	0.34	0.47	0.46	

^a^ *N* = number of sequences; *k*, number of polymorphic sites; *h*, number of haplotypes; *H*_*d*_, haplotype diversity; and *π*, nucleotide diversity

^b^ Rarefaction estimates of mean allelic richness for twelve loci based on minimum sample size of 3

^c^ Rarefaction estimates of mean private allelic richness for twelve loci based on minimum sample size of 3

Haplotype networks for eleven nuclear loci did not exhibit comparable fixed differences between the NEPR and the southern (SEPR + PAR) groups (Fig. 2a). Instead, allelic frequencies varied among populations in a geographically structured manner (Fig. 2b), for which the blue-shaded (NEPR) alleles yielded to green-shaded (SEPR) alleles, which yielded, in turn, to red-shaded (PAR) alleles. Although the frequencies varied among populations, they did not show congruent gradient among different loci. *SAHH* and *AP_NC43* had the simplest haplotype networks, with a single dominate allele (*q*_i_ ≥ 0.7) and variants that differed by only one or two mutations. *AP_NC1* also had a single dominant allele, but some of NEPR variants (blue) differed by as many as 11 mutational steps. The remaining eight loci exhibited more diverse networks. In general, the NEPR samples had more singletons and rare alleles than SEPR and PAR samples. These regional differences were reflected in estimates of genetic diversity (Table 2). Aside from the regional effects, differences in diversity also existed among loci. For example, *AP_NC32* exhibited the greatest overall nucleotide diversity (*π* = 0.0130, Table 2), whereas *AP_NC43* had the smallest (*π* = 0.0014). Haplotype diversity (*H*_*d*_) ranged from 0.44 (*SAHH*) to 0.87 (*mtCOI*) for the protein-coding markers, and from 0.41 (*AC_NC1*) to 0.87 (*AC_NC22*) for the non-coding markers.

Estimators of genetic diversity declined with southern latitudes (Table 2). For example, haplotype diversity (*H*_*d*_, *r* = −0.893, *P* < 0.001), rarefaction estimates of allelic richness (*r* = −0.912, *P* < 0.001), and private alleles (*r* = −0.867, *P* = 0.001) all decreased with southern latitudes. Estimates of allelic richness and of private alleles are correlated (*r* = 0.909, *P* < 0.001). Although *H*_*d*_ is expected to be more sensitive to the evenness of allelic frequencies, in this case *H*_*d*_ is almost perfectly correlated with richness (*r* = 0.997, *P* < 0.001). Tectonic spreading rates for the sampled localities (Fig. 1) also increased with southern latitudes (*r* = 0.721, *P* = 0.019); consequently, spreading rates also were inversely correlated with genetic diversity *H*_*d*_ (*r* = −0.724, *P* = 0.018) and richness (*r* = −0.740, *P* = 0.014).

### Geographical structure

**Fig. 3:**
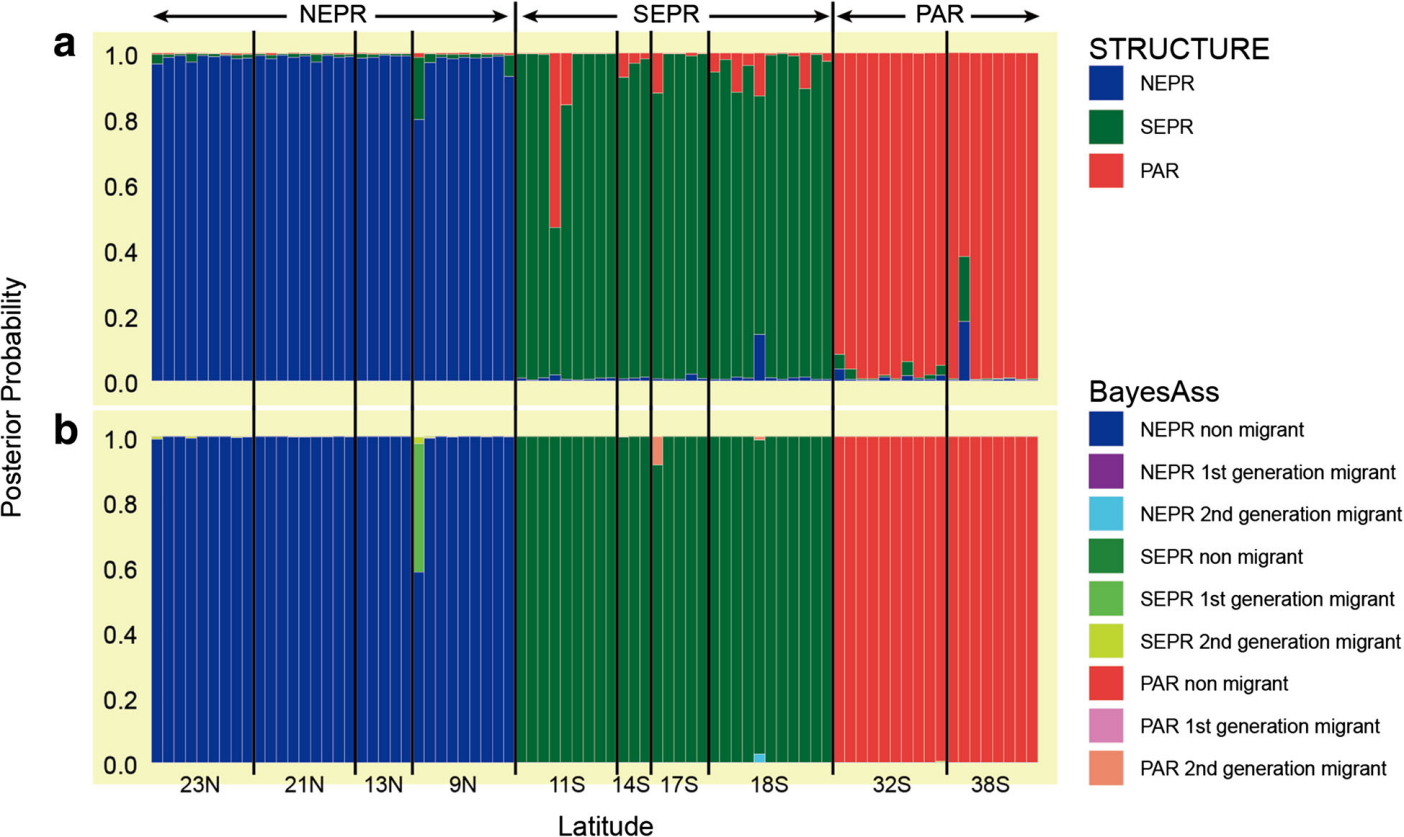
Multi-locus genotypic assignments for nuclear markers. **a** Structure analysis with *K* = 3. Vertical bars indicate probability that an individual could be assigned to the NEPR (*blue*), SEPR (*green*) and PAR (*red*) clusters. **b** BayesAss assignments of migrant ancestries. Color codes indicate posterior probabilities that individuals had native versus immigrant ancestries

The Structure analysis identified three geographical clusters (*K* = 3, Δ*K* = 1431.57): NEPR (23N, 21N, 13N, and 9N); SEPR (11S, 14S, 17S, and 18S); and PAR (32S and 38S), north to south in order (Fig. 3a, Additional file 2: Table S2). Samples from the recently discovered vents at 23N and 38S clustered with those from the neighboring NEPR and PAR vents, respectively. The Structure analysis identified very limited evidence for mixed ancestries within the three clusters, without any noticeable gradient around the geographical boundaries. Bayesass is capable of identifying recent immigrants from the other clusters (Fig. 3b) but contemporary immigration appeared to be very limited. Only two putative second-generation immigrants were identified with very low posterior probabilities: the light green individual in NEPR (*PP* = 0.40); and the pink individual in SEPR (*PP* = 0.087).

AMOVA identified hierarchical partitioning of genetic variation for the 11 nuclear loci: 22.7 % among the NEPR, SEPR and PAR regions, 2.6 % among samples within regions, and 74.7 % among individuals within samples. For *mtCOI*, more of the variation (52.8 %) existed among regions, 0.6 % among samples within regions, and 46.6 % among individuals within samples. Mantel tests identified significant correlations between genetic and geographic distances (Table 3, Additional file 3: Figure S1: for nuclear genes, *r* = 0.463, *P* = 0.002; and for *mtCOI*, *r* = 0.705, *P* = 0.003). However, assignment of each sample to its respective geographical region (i.e. strata), and application of the stratified Mantel test revealed no significant residual correlation with geographical distance (*P* = 0.120 for nuclear genes; and *P* = 0.626 for *mtCOI* gene).

**Table 3:** Pairwise differentiation (*F*_ST_) for *mtCOI* (above diagonal) and for nuclear genes (below diagonal)

	23N	21N	13N	9N	11S	14S	17S	18S	32S	38S
23N		−0.008	0.147	0.058	**0.539**	**0.399**	**0.444**	**0.571**	**0.571**	**0.542**
21N	−0.012		−0.073	−0.063	**0.576**	**0.442**	**0.481**	**0.607**	**0.607**	**0.581**
13N	−0.007	0.019		−0.069	**0.720**	**0.611**	**0.628**	**0.749**	**0.748**	**0.738**
9N	−0.023	−0.011	−0.034		**0.542**	**0.410**	**0.453**	**0.572**	**0.573**	**0.546**
11S	**0.136**	**0.152**	**0.086**	**0.119**		−0.136	0.030	0.002	0.120	0.028
14S	0.033	**0.060**	0.016	0.015	0.006		−0.060	−0.011	0.222	0.178
17S	0.031	0.041	0.006	0.019	0.023	−0.011		0.050	**0.209**	0.140
18S	**0.089**	**0.102**	**0.075**	**0.094**	0.025	−0.004	−0.010		**0.231**	0.138
32S	**0.122**	**0.139**	**0.098**	**0.092**	**0.164**	0.085	**0.104**	**0.151**		−0.079
38S	**0.210**	**0.221**	**0.165**	**0.166**	**0.243**	**0.233**	**0.208**	**0.253**	**0.081**	

Bold cases represent statistical significance at *α* = 0.05

### Isolation with Migration (IMa2) analyses

**Fig. 4:**
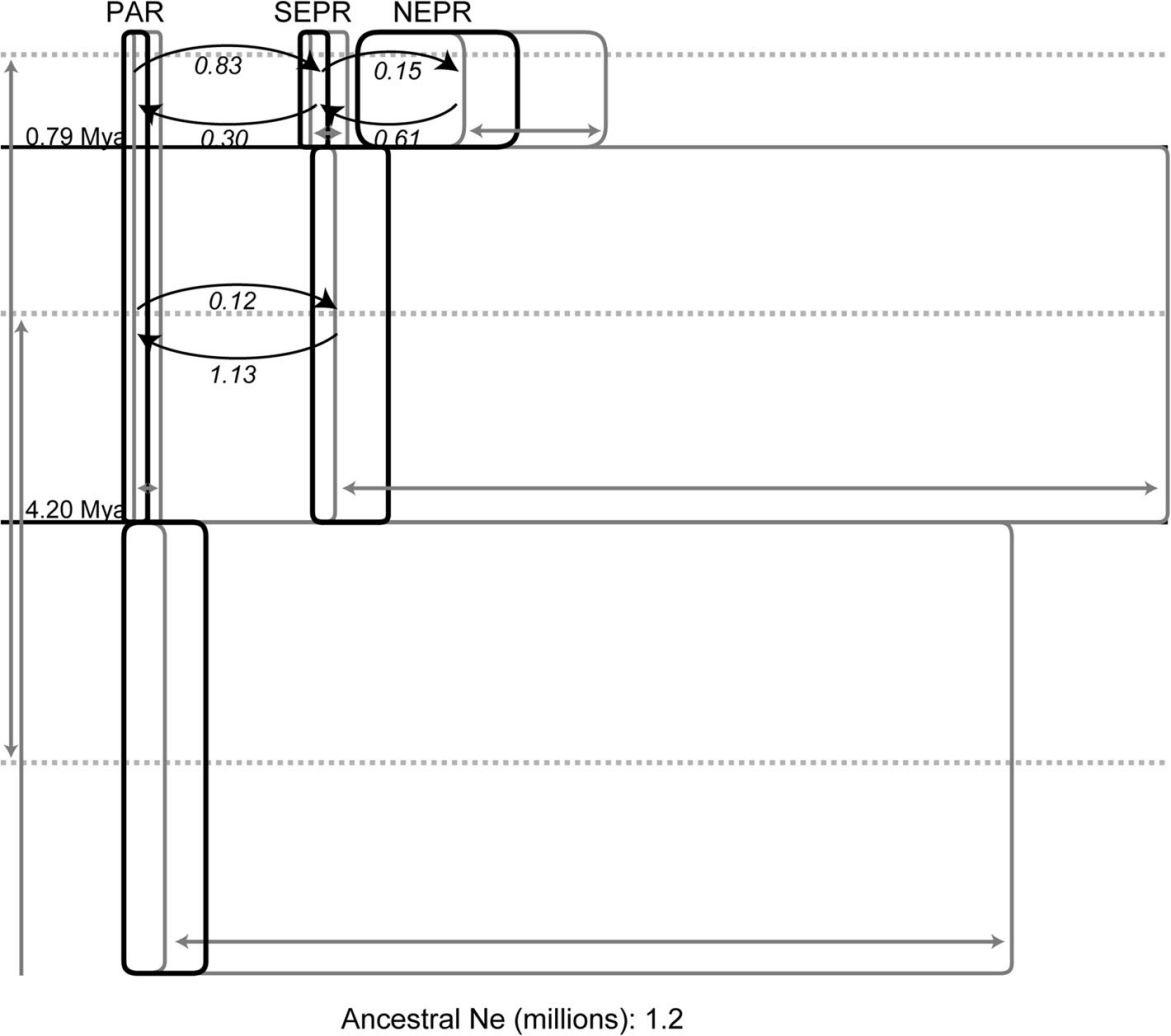
Diverging processes of *A. pompejana* in the EPR estimated with IMa2. The width of the black boxes represents effective population sizes of each population and each ancestral population, and the width of the *gray* boxes represents the 95 % highest posterior density (HPD) intervals of them. The *horizontal lines* represent divergence time between populations. The *curved arrows* represent migration rate (2 *Nm*) between populations forward in time. The *gray* arrows represent 95 % HPD intervals of each demographic parameter. Note that the upper bound of 95 % HPD of the divergence time between PAR and its sister northern group about 4.2 Mya is not shown

Analysis of a three-population IMa2 model (Fig. 4, Additional file 4: Table S3) detected evidence that southward migration (from NEPR into SEPR, 2*N*_s_ *m*_s_ = 0.61) exceeded northward migration (2*N*_n_ *m*_n_ = 0.15). A series of nested model tests on this observation resulted in statistically supports for the presence of gene flow (Additional file 4: Table S4 and S5). However, these tests could not reject other alternative hypotheses: an equal migration rate for the southward and northward migrations, and unidirectional migration. Analyses also detected greater southward migration from the SEPR/NEPR ancestral population into the PAR population (2 *Nm* = 1.13 vs. 0.12), although unidirectional migration was not rejected (Fig. 4, Additional file 4: Table S4 and S5). However, gene flow from PAR into SEPR was greater after the split between NEPR and SEPR about 0.79 Mya (95 % HPD: 0.07–6.67 Mya) (2 *Nm* = 0.30 vs. 0.83). This northward direction was statistically supported by the log-likelihood ratio test. The estimated time of population splitting between the SEPR-PAR pair was much older, ~4.20 Mya (95 % HPD: 2.42–33.42 Mya) (Fig. 4). Estimates of effective population sizes (*N*) can be ranked in the following order: NEPR > SEPR > PAR.

### Demographic stability

Tajima’s *D* and Fu’s *F*_S_ statistics can be sensitive indicators of demographic processes [32, 33]. Altogether, 120 tests of these metrics for each locus in population resulted in only two significantly negative values, following Bonferroni corrections for multiple tests (Additional file 5: Table S6). However, samples sizes per gene per locality were limited and statistical power was low. Thus, we pooled individual samples within the NEPR, SEPR and PAR regions. Pooling samples might bias *D* and *F*_S_ metrics in a positive direction if samples are genetically heterogeneous within each region, but the vast majority of estimates were still negative, and only a few were statistically significant, following Bonferroni corrections (Additional file 5: Table S7). None of the positive estimates were significant. Consequently, no substantive evidence for recent demographic bottlenecks or expansions was found.

## Discussion

Deep-sea expeditions conducted during 2005 and 2015 obtained samples that extend the known range of *Alvinella pompejana* northward by 288 km to the Alarcón Basin in the Gulf of California (23°N latitude) and southward by 666 km to a northeastern segment of the Pacific Antarctic Ridge (38°S latitude). Multi-locus genotypes of individuals sampled from ten localities distributed across this range clustered into three metapopulation segments (Fig. 3). The Equator separates the NEPR and SEPR segments, and the Easter Microplate region separates the SEPR and PAR segments (Fig. 1). Large portions of the DNA sequence diversity in *A. pompejana* resided in the variation among individuals within each sample location: 46.6 % for *mtCOI* and 74.7 % for nuclear genes. Most of the remaining diversity resided in the differences among samples from the three regions: 52.8 % for *mtCOI* and 22.7 % for nuclear genes. Very small portions of the diversity resided in the differences among sample localities within regions: only 0.6 % for *mtCOI* and 2.6 % for nuclear genes. This lack of within-region differentiation reflected very high rates of gene exchange along each ridge axis (discussed below). Dispersal along contiguous segments of the three ridge axes appeared to be relatively unimpeded, despite high bottom-currents and distances of several hundred kilometers between active vents [20, 53]. Developmental arrest of its embryos and delayed metamorphosis undoubtedly plays a significant role in *A. pompejana*’s capacity for long-distance dispersal [54]. Disconnection and reconnection of active vent habitats over time due to frequent shifts of the magma supply might also contribute to the genetic homogeneity of *A. pompejana* populations distributed along a ridge axis [55].

Estimates of genetic differentiation (*F*_ST_ values) increased significantly with geographical distances among the sample locations (Table 3; Additional file 3: Figure S1). This apparent Isolation-by-Distance (IBD) pattern was previously inferred to result from stepping-stone dispersal [4]. We observed the same pattern for nuclear and mitochondrial genes, but application of stratified Mantel tests, as developed by Meirmans [56], revealed that the pattern resulted from hierarchical subdivision. IBD-like patterns can result from a variety of processes including hierarchical structure, geographical selection gradients, secondary intergradation and range expansions (e.g., [57–59]).

### Spreading rates, disturbance and genetic diversity

Tectonic spreading rates have been interpreted as surrogates for frequencies of habitat turnover due to local extirpations from tectonic and volcanic events and colonizations of new or “reborn” habitats [1, 60]. Increased frequencies of habitat turnover were expected to reduce genetic diversity within localities and increase the homogeneity among the localities [61, 62]. As predicted, haplotype diversity in the *A. pompejana* samples decreased significantly as tectonic spreading rates increase in southern latitudes (*r* = −0.724, *P* = 0.018). The PAR and SEPR axes exhibit “superfast” spreading rates of 141–151 mm/yr [63]. Reduced genetic diversity of these southern populations of *A. pompejana* and the co-distributed siboglinid tubeworm, *Riftia pachyptila*, correspond with the rapid cycles of habitat extinction and rebirth in this region [22]. Such demographic instability is also expected to leave other genetic footprints. Previous population genetic studies of *A. pompejana* reported evidence for recent demographic expansion within individual populations [18, 20]. In contrast, the present estimates of Tajima’s *D* and Fu’s *F*_S_ from a larger sample of genes did not corroborate these conclusions (Additional file 5: Tables S6 and S7). The present gene networks (Fig. 2a) do not exhibit the star-like clusters of haplotypes typically associated with such events [18].

The IMa2 analyses provide additional information about demographic processes. The NEPR cluster appears to have increased in size from the hypothetical ancestral population, whereas the southern SEPR and PAR clusters may have become smaller (Fig. 4). The trend of decreasing population size with southern latitudes (NEPR > SEPR > PAR) corresponded with increasing tectonic spreading rates (Fig. 1). Nonetheless, relatively large effective population sizes of each regional group (Additional file 4: Table S3; Additional file 5: Table S8) suggest that *A. pompejana* has maintained high site occupancy within each region. Indeed, *A. pompejana* is one of the genetically most diverse vent invertebrates studied to date [1]. This pioneer species is among the first animals to colonize nascent hydrothermal vents [64]. It can persist in hydrothermal flows approaching 50 °C [11] that would appear to exclude potential competitors like the siboglinid tubeworms *Riftia pachyptila* and *Tevnia jerichonana*, and the bivalves *Bathymodiolus thermophilus* and *Calyptogena magnifica*. Coupled with its exceptional colonization abilities, much lower rates of local extirpation probably explain the ability of this species to retain such higher levels of genetic diversity.

### The equatorial boundary

A deep strong eastward current crossing the East Pacific Rise at the Equator generates northern and southern gyres [65] and might impede along-axis dispersal of vent species that produce pelagic larvae. However, surface currents are unlikely to affect *A. pompejana*, a species that produces negatively buoyant larvae with benthic dispersal [18, 64]. Gaps in the spatial or temporal frequency of hydrothermal habitats are expected to disrupt the dispersal of vent species [4]. Active vents supporting *A. pompejana* have not been reported within 900 km of the Galápagos Triple Junction region (Fig. 1), creating a large contemporary gap in the distribution of this species. Nonetheless, we cannot exclude the possibility that the Triple Junction region hosts active vents, because this region is not well explored. Furthermore, the age of this putative gap is unknown. It might be coincide with formation of the Hess Deep formed about 1 Mya [66]. Auspiciously, our estimated time of separation between NEPR and SEPR population segments of *A. pompejana* was about 0.79 million years ago (95 % HPD: 0.07–6.67 Mya; Fig. 4, Additional file 4: Table S3). Using similar methods, Plouviez et al. [20] estimated a slightly older time of separation, 1.2–1.3 Mya for *A. pompejana*. This slight difference must be due to different data sets and substitution rates used in both studies.

The Equatorial region creates a semipermeable barrier to dispersal by *A. pompejana* and several other vent species. The IMa2 analysis (Fig. 4) provided evidence for weak but statistically significant gene flow across the filter (Fig. 4, Additional file 4: Table S4 and S5). This pattern was also evident in the analysis of BayesAss which exhibited a few candidates of recent immigration (Fig. 3). Nevertheless, the extent of gene flow does not seem to be sufficient to prevent *A. pompejana* from diverging across the Equatorial filter. The siboglinid tubeworms *Tevnia jerichonana* and *Riftia pachyptila* also exhibit evidence for partial isolation across this boundary, but their subdivision might be due to historical range expansions or recolonizations of the SEPR axis from NEPR sources [21, 22]. Several gastropod limpets also clustered into groups separated by this boundary [19, 67]. In contrast, the polychaete annelids *Branchipolynoe symmytilida* and *Hessiolyra bergi*, and bivalve mussel *Bathymodiolus thermophilus* exhibit no substantive genetic differentiation across this boundary [18, 19, 23].

### The Easter Microplate boundary

Formation of the Easter Microplate resulted in severe topographic changes that originated 2.5–5.3 Mya [68, 69]. Our estimates of the time of splitting between the PAR and SEPR population segments were not well resolved, but the process of divergence may be at least 3.3 million year (My) old (Fig. 4, Additional file 5: Figure S2 and Table S8). Moreover, Won et al. [2] hypothesized that strong currents create a contemporary barrier to dispersal of deep-sea species across this boundary. Geostrophic models and empirical evidence indicate such a presence of strong cross-axis currents in the 22–25°S region of the East Pacific Rise [70, 71]. Recently, McGillicuddy et al. study [72] provides more information on the characteristics of larval dispersal under more realistic hydrographic influences along the ridge axis of EPR. Using simulation of particle transporting along a virtual mid-ocean ridge of the EPR, they found that the dispersal distance of numerical larvae decreases as the height where they stay above the ocean bottom increased. The result arises from the fact that as larvae closer to the bottom tend to be more influenced by strong currents along the flanks of ridge, they are transported farther than other ones staying higher from the bottom. The larval transporting simulation clearly showed that deep-sea currents on the ridge axis influence on the direction and distance of larval dispersal. Marsh et al. [7] also reported a long-distance (~100 km) dispersal potential of the larvae of tubeworm, *R. pachyptila*, when it met a favorable deep-sea flow along the EPR. Otherwise, most proportion of them might be retained near to the source population. It is manifest that the hydrodynamic effects on the larval dispersal are important and can contribute to shape population genetic structures of vent animals. However, the structures are also products of other extrinsic and intrinsic factors and their interactions, including ridge geomorphology, temporal stability of vents, larval developments and behaviors [1].

The formation of Easter Microplate and its associated deep-sea currents appear to be the primary cause for the parallel divergence observed in several vent species co-distributing around the Easter Microplate boundary. Like *A. pompejana*, the siboglinid tubeworm *Tevnia jerichonana* also exhibits evidence for partial isolation across this boundary [21]. The vent mussels *Bathymodiolus thermophilus* and *B. antarcticus* meet and hybridize at this boundary [23]. Sister species of vent crabs *Bythograea laubieri* and *B. vrijenhoeki* also separate across this boundary [73, 74].

## Conclusions

The geographical distribution of genetic diversity of *A. pompejana* is consistent with a metapopulation model that predicts a decline in diversity along superfast-spreading axes due to frequent local extinctions and rebirths of vent habitats [1, 60]. Unique life history characteristics of *Alvinella* worms, a pioneering colonizer of hydrothermal vents, contribute to its high dispersal capability along contiguous segments of the NEPR, SEPR and PAR axes, but dispersal between the three regions is limited. A large portion of the total genetic diversity (22.7 %, nuclear genes; and 52.8 %, *mtCOI* gene) was partitioned among three geographical regions. Maximum likelihood estimates of divergence times suggest that subdivision originated ~1 Mya across the Equator and 2.5–5.3 Mya across the Easter Microplate boundary. Nonetheless, low degrees of gene flow (2 *Nm* < 1) appeared to maintain some genetic continuity across both boundaries.

## Additional files

Additional file 1: Table S1. Primer pairs for nested PCR of twelve genetic loci of *Alvinella pompejana*. (DOCX 21.3 kb)
Additional file 2: Table S2. Likelihood values of Structure. (DOCX 17.2 kb)
Additional file 3: Figure S1. Relationship between genetic differentiation (*F*_ST_) and geographical distance (km): *F*_ST_ of nuclear genes (black) and *F*_ST_ of *mtCOI* gene (white). Mantel’s *r* and significance of correlation (*P*-value) is listed in a box. (DOCX 199 kb)
Additional file 4:
**Table S3**. Isolation with Migration 3-population analyses (NEPR, SEPR and PAR). **Table S4**. Log-likelihood ratio tests for nested models of migration in Isolation with Migration 3-population analyses (NEPR, SEPR and PAR). **Table S5**. Log-likelihood ratio tests between a model of absence of gene flow and the other nested models of migration. (DOCX 39.5 kb)
Additional file 5: Table S6. Tajima’s *D* and Fu’s *F*_S_ of each locus in each sample. **Table S7**: Tajima's *D* and Fu’s *F*_S_ of each genetic locus across three geographical groups. **Table S8**. Isolation with Migration 2-population analyses. Estimated effects of Equatorial and Easter Microplate boundaries on demographic parameters. Maximum likelihood estimates (MLE) of six demographic quantities. **Figure S2**. Posterior probability estimates for population demographic quantities of the model parameters of IMa2 2-population analyses. Posterior probability curves of divergence time (*t*) (A), migration rates (2*Nm*) (B), and effective population sizes (N) (C) between NEPR and SEPR are illustrated on the upper panel, and same posterior curves for the quantities between SEPR and PAR are shown in (D), (E), and (F). The geographical groups of *N* in figures (C) and (F) are represented as subscripts: n, NEPR; s, SEPR; p, PAR; and a, ancestral population. (DOCX 309 kb)

## Abbreviations

AMOVA: Analysis of molecular variance
GAR: Galápagos Rift
*GlobX*: Globin X
HOV: Human occupied vehicle
HPD: Highest posterior density
IBD: Isolation-by-distance
IMa: Isolation with migration analyses
LD: Linkage disequilibrium
*mtCOI*: Mitochondrial cytochrome-c-oxidase subunit-I
Mya: Million years ago
NEPR: Northern East Pacific Rise
PAR: Pacific Antarctic Ridge
*PGM*: Phosphoglucomutase
ROV: Remotely operated vehicle
*SAHH*: S-adenosylhomocysteine hydrolase
SEPR: Southern East Pacific Rise
