# Supplementary material for: Population subdivision of hydrothermal vent polychaete Alvinella pompejana across equatorial and Easter Microplate boundaries
Source: BMC Evol Biol. 2016 Oct 28;16:235. doi: 10.1186/s12862-016-0807-9 (PMC5084463; doi:10.1186/s12862-016-0807-9)
Supplement: Additional file 1: Table S1. — Primer pairs for nested PCR of twelve genetic loci of Alvinella pompejana. (DOCX 21.3 kb) [file 12862_2016_807_MOESM1_ESM.docx]

**Additional file 1**

**Table S1.** Primer pairs for nested PCR of twelve genetic loci of *Alvinella pompejana*.

| Locus |  | Primer sequence (5'-3') |  | Primer sequence (5'-3') | T_m_(°C)^*^ | L^1^ | L^2^ |
| --- | --- | --- | --- | --- | --- | --- | --- |
| *mtCOI* | F | GGTCAACAAATCATAAAGATTGG | R | TAAACTTCAGGGTGACCAAAAAATCA | 45 | 513 | 513 |
|  | 2F | - | 2R | TAGTAGCATAGTAATGGCAC | 55 |  |  |
| *Globx* | F | AGAGAAGCGGTCGTGGAGAG | R | AGCATACCGAATTGAATCTA | 55 | 364 | 322 |
|  | 2F | TGGAAGGAAATCTGTAAAGA | 2R | ATGTTGTAATCAACTGTATA | 52 |  |  |
| *PGM* | F | CAATGGTAACAGCTGAATGT | R | TGGAATCTGGATCTACTAGT | 50 | 326 | 326 |
|  | 2F | TATTTTTCAAATGTGATCTA | 2R | ACTTTGCTCTTTAAATATCA | 50 |  |  |
| *SAHH* | F | ATGGGACATCCAAGCTTCGT | R | TTGTCAGTCTGACACCAAGA | 55 | 402 | 402 |
|  | 2F | TGTCAAACTCGTTCACGAAC | 2R | TTCAAGATGTGCAGCGGCCA | 55 |  |  |
| *AP_NC1* | F | AATATGACGTCATAAATG | R | GTAGTTTAGGTTGGAAGG | 45 | 380 | 201 |
|  | 2F | - | 2R | TTTTGCTCTCTGATCTTG | 50 |  |  |
| *AP_NC3* | F | GAGCACGCACTTAAGTCC | R | ACTCTTAACGTCCACGCC | 50 | 375 | 296 |
|  | 2F | - | 2R | GATACGACCGGCGCCTGT | 55 |  |  |
| *AP_NC8* | F | CTTGAAATCCTTCAAAGA | R | CCTGCAAAATGGCACTGC | 50 | 351 | 184 |
|  | 2F | - | 2R | TGTCGGATTTATCTGTTT | 50 |  |  |
| *AP_NC20* | F | ACAATCGTACCACATAAA | R | GATGTTGAAGTCCCAAGC | 50 | 350 | 175 |
|  | 2F | - | 2R | TGACTACCACACACCTCG | 55 |  |  |
| *AP_NC22* | F | CAGATCTCCTCCAGCAAT | R | TTCAGTGAACGGGGCTTA | 55 | 351 | 333 |
|  | 2F | - | 2R | GTATTGTCGCCGTGTATA | 55 |  |  |
| *AP_NC28* | F | TAAACAGCACAAGACGAG | R | GTAGCGTCGTCGACATAA | 50 | 376 | 246 |
|  | 2F | - | 2R | TCCGGTTGTCGCTCTGTG | 58 |  |  |
| *AP_NC32* | F | TATAGTGCAGCTCTGCTT | R | GTTGTATAAAGTTGTGGC | 50 | 365 | 267 |
|  | 2F | - | 2R | AGGTACTCGTTTTTAAAG | 50 |  |  |
| *AP_NC43* | F | GGAAAGTAGCCACATTTC | R | ATTATGTTCCACTTGTTC | 48 | 353 | 353 |
|  | 2F | - | 2R | TTCATGGTGTCTTAATGT | 50 |  |  |

^*^ *T_m_* indicates annealing temperature of PCR.

^1^ Length of the sequence prior to SITES analysis.

^2^ Length of the sequence after SITES analysis.
