# Supplementary material for: Population subdivision of hydrothermal vent polychaete Alvinella pompejana across equatorial and Easter Microplate boundaries
Source: BMC Evol Biol. 2016 Oct 28;16:235. doi: 10.1186/s12862-016-0807-9 (PMC5084463; doi:10.1186/s12862-016-0807-9)
Supplement: Additional file 2: Table S2. — Likelihood values of Structure. (DOCX 17.2 kb) [file 12862_2016_807_MOESM2_ESM.docx]

**Additional file 2**

**Table S2.** Likelihood values of STRUCTURE

| *K* | Reps | Mean Ln P(*K*) | Stdev LnP(*K*) | Ln'(*K*) | \|Ln''(*K*)\| | Delta *K* |
| --- | --- | --- | --- | --- | --- | --- |
| 1 | 5 | -3052.20 | 0.72 | NA | NA | NA |
| 2 | 5 | -2675.28 | 11.43 | 376.92 | 114.64 | 10.03 |
| **3** | **5** | **-2413.00** | **0.31** | **262.28** | **441.24** | **1431.57** |
| 4 | 5 | -2591.96 | 12.83 | -178.96 | 178.86 | 13.94 |
| 5 | 5 | -2592.06 | 98.66 | -0.10 | 86.72 | 0.88 |
| 6 | 5 | -2505.44 | 12.03 | 86.62 | 201.32 | 16.74 |
| 7 | 5 | -2620.14 | 20.69 | -114.70 | 35.34 | 1.71 |
| 8 | 5 | -2699.50 | 38.59 | -79.36 | 4.80 | 0.12 |
| 9 | 5 | -2783.66 | 25.98 | -84.16 | 31.00 | 1.19 |
| 10 | 5 | -2836.82 | 55.97 | -53.16 | NA | NA |
