# Supplementary material for: Population subdivision of hydrothermal vent polychaete Alvinella pompejana across equatorial and Easter Microplate boundaries
Source: BMC Evol Biol. 2016 Oct 28;16:235. doi: 10.1186/s12862-016-0807-9 (PMC5084463; doi:10.1186/s12862-016-0807-9)
Supplement: Additional file 3: Figure S1. — Relationship between genetic differentiation (F ST) and geographical distance (km): F ST of nuclear genes (black) and F ST of mtCOI gene (white). Mantel’s r and significance of correlation (P-value) is listed in a box. (DOCX 199 kb) [file 12862_2016_807_MOESM3_ESM.docx]

**Additional file 3**


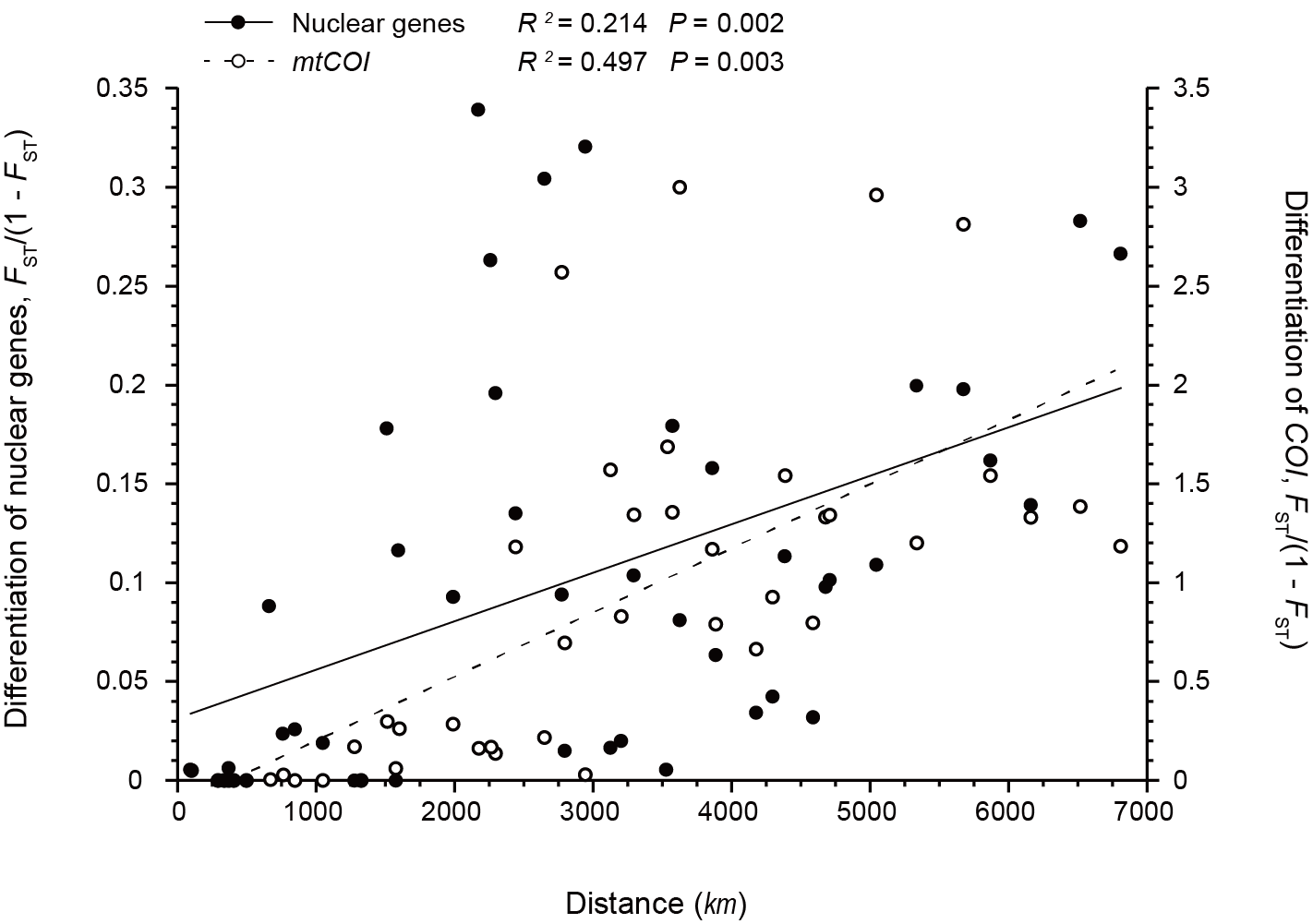


**Figure S1.** Relationship between genetic differentiation (*F*_ST_) and geographical distance (km): *F*_ST_ of nuclear genes (black) and *F*_ST_ of *mtCOI* gene (white). Mantel’s *r* and significance of correlation (*P*-value) is listed in a box.
