# Supplementary material for: Population subdivision of hydrothermal vent polychaete Alvinella pompejana across equatorial and Easter Microplate boundaries
Source: BMC Evol Biol. 2016 Oct 28;16:235. doi: 10.1186/s12862-016-0807-9 (PMC5084463; doi:10.1186/s12862-016-0807-9)
Supplement: Additional file 4: — Table S3. Isolation with Migration 3-population analyses (NEPR, SEPR and PAR). Table S4. Log-likelihood ratio tests for nested models of migration in Isolation with Migration 3-population analyses (NEPR, SEPR and PAR). Table S5. Log-likelihood ratio tests between a model of absence of gene flow and the other nested models of migration. (DOCX 39.5 kb) [file 12862_2016_807_MOESM4_ESM.docx]

**Additional file 4**

**Table S3.** Isolation with Migration 3-population analyses (NEPR, SEPR, and PAR).

| Three population model | | | |
| --- | --- | --- | --- |
|  | MLE | L^1^ | U^1^ |
| N_n_^2^ | 1.64 | 1.09 | 2.58 |
| N_s_ | 0.25 | 0.12 | 0.52 |
| N_p_ | 0.17 | 0.08 | 0.35 |
| N_a2_ | 0.81 | 0.20 | 9.43 |
| N_a1_ | 1.21 | 0.62 | 10.01 |
| 2N_n_m_n>s_^3^ | 0.15 | 0.00 | 2.15 |
| 2N_s_m_s>n_^4^ | 0.61 | 0.08 | 1.19 |
| 2N_s_m_s>p_^5^ | 0.83 | 0.37 | 1.63 |
| 2N_p_m_p>s_^6^ | 0.30 | 0.03 | 0.71 |
| 2N_a2_m_a2>p_^7^ | 0.12 | 0.00 | 152.40 |
| 2N_p_m_p>a2_^8^ | 1.13 | 0.00 | 3.75 |
| t_0_ (Mya) ^9^ | 0.79 | 0.07 | 6.67 |
| t_1_ (Mya) ^10^ | 4.20 | 2.42 | 33.42 |

^1^ Lower (L) and upper (U) bounds for 95% highest posterior density (HPD).

^2^ Effective population size of NEPR as a unit of one million individuals. The subscript ‘n’ stands for the geographic group, NEPR. Likewise, the subscripts, s, p, and a, stand for the other geographic groups, SEPR, PAR, and ancestral population, respectively. Three populations model has two ancestral populations; a1 stands for effective populations size of ancestor group of three geological groups and a2 stands for effective population size of ancestor group of NEPR and SEPR groups.

^3^ m_n>s_, migration rate into NEPR from SEPR forward in time.

^4^ m_s>n_, migration rate into SEPR from NEPR forward in time.

^5^ m_s>p_, migration rate into SEPR from PAR forward in time.

^6^ m_p>s_, migration rate into PAR from SEPR forward in time.

^7^ m_a2>p_, migration rate into ancestor group of NEPR and SEPR from PAR forward in time.

^8^ m_p>a2_, migration rate into PAR from ancestor group of NEPR and SEPR forward in time.

^9^ t_0_, Splitting time into NEPR and SEPR in million years.

^10^ t_1_, Splitting time into PAR and ancestor group of NEPR and SEPR in million years.

**Table S4.** Log-likelihood ratio tests for nested models of migration in Isolation with Migration 3-population analyses (NEPR, SEPR, and PAR).

| Model Description | log(P) | df | -2*Δ* | *P* |
| --- | --- | --- | --- | --- |
| 0 ALL MIGRATION PARAMETERS | -1.030 | - | - |  |
| 1 *θ*_n_ ^1^*θ*_s_ *θ*_a2_ *m*_n>s_^2^ = *m*_s>n_ | -2.134 | 1 | 2.209 | 0.137 |
| 2 *θ*_n_ *θ*_s_ *θ*_a2_ *m*_n>s_ = 0, *m*_s>n_ | -2.028 | 1* | 1.996 | 0.079 |
| 3 *θ*_n_ *θ*_s_ *θ*_a2_ *m*_n>s_, *m*_s>n_ = 0 | -1.897 | 1* | 1.736 | 0.094 |
| 4 *θ*_n_ *θ*_s_ *θ*_a2_ *m*_n>s_ = 0, *m*_s>n_ = 0 | -3.778 | 2^#^ | 5.497 | **-** |
| 5 *θ*_s_ *θ*_p_ *θ*_a1_ *m*_s>p_ = *m*_p>s_ | -3.059 | 1 | 4.059 | **0.044** |
| 6 *θ*_s_ *θ*_p_ *θ*_a1_ *m*_s>p_ = 0, *m*_p>s_ | -218.100 | 1* | 434.100 | **0.000** |
| 7 *θ*_s_ *θ*_p_ *θ*_a1_ *m*_s>p_, *m*_p>s_ = 0 | -2.306 | 1* | 2.553 | 0.055 |
| 8 *θ*_s_ *θ*_p_ *θ*_a2_ *m*_s>p_ = 0, *m*_p>s_ = 0 | -561.300 | 2^#^ | 1120.000 | **-** |
| 9 *θ*_p_ *θ*_a2_ *θ*_a1_ *m*_p>a2_ = *m*_a2>p_ | -3.455 | 1 | 4.852 | **0.028** |
| 10 *θ*_p_ *θ*_a2_ *θ*_a1_ *m*_p>_a_2_ = 0, *m*_a2>p_ | -1.974 | 1* | 1.888 | 0.085 |
| 11 *θ*_p_ *θ*_a2_ *θ*_a1_ *m*_p>_a_2_, *m*_a2>p_ = 0 | -1.030 | 1* | 0.000 | 1.000 |
| 12 *θ*_p_ *θ*_a2_ *θ*_a1_ *m*_p>_a_2_ = 0, *m*_a2>p_ = 0 | -8.457 | 2^#^ | 14.860 | **-** |

^*^ Test distribution of 2LLR is a mixture. The p-value was divided two according to Jody and Nielsen, 2007 [[1](#_ENREF_1)].

^#^ When the null model is true and has two parameters fixed at the boundary of the parameter space, the expected asymptotic distribution of -2*Δ* (log-likelihood ratio) is a mixture of $\chi_{0}^{2}$, $\chi_{1}^{2}$, and $\chi_{2}^{2}$ with probability of 1/2 - *p*, 1/2, and *p*, respectively [[2](#_ENREF_2)]. According to Self and Liang [[see the case 7 in this reference, 2](#_ENREF_2)], the function of mixing probability, *p*, takes a complex form. Thus we did not calculate it for the nested models where both migration rates were fixed at zero.

^1^ *θ*_n_: effective population size. The subscript stands for geographic group as written in Table S4.

^2^ *m*_x>y_: migration rate into ‘x’ group from ‘y’ forward in time as written in Table S3.

Bold cases represent statistical significance at *α* = 0.05.

**Table S5.** Log-likelihood ratio tests between a model of absence of gene flow and the other nested models of migration

| H0^1^ | H1^2^ | -2*Δ* | *P* | d.f. |
| --- | --- | --- | --- | --- |
| M4^#^ | M1 | 3.288 | **0.03485** | 1* |
| M4 | M2 | 3.501 | **0.03065** | 1* |
| M4 | M3 | 3.761 | **0.02620** | 1* |
| M8 | M5 | 1115.941 | **0.00000** | 1* |
| M8 | M6 | 685.900 | **0.00000** | 1* |
| M8 | M7 | 1117.447 | **0.00000** | 1* |
| M12 | M9 | 10.008 | **0.00075** | 1* |
| M12 | M10 | 12.972 | **0.00015** | 1* |
| M12 | M11 | 14.860 | **0.00005** | 1* |

^*^ Test distribution of 2LLR is a mixture. The *p*-value was divided two according to Jody and Nielsen, 2007 [[1](#_ENREF_1)].

^1^ Null hypothesis: absence of gene flow in both directions.

^2^ Alternative hypothesis

^#^ M indicates nested models of migration, and the number after “M” is consistent with the number in front of the nested model test in the Table S5. For example, M4 indicates the forth nested model of Table S5, *θ*_n_ *θ*_s_ *θ*_a2_ *m*_n>s_ = 0, *m*_s>n_ = 0.

Reference

1. Hey J, Nielsen R: **Integration within the Felsenstein equation for improved Markov chain Monte Carlo methods in population genetics**. *Proceedings of the National Academy of Sciences of the United States of America* 2007, **104**(8):2785-2790.

2. Self SG, Liang K-Y: **Asymptotic properties of maximum likelihood estimators and likelihood ratio tests under nonstandard conditions**. *Journal of American Statistical Association* 1987, **82**(398):605-610.
