# Supplementary material for: Population subdivision of hydrothermal vent polychaete Alvinella pompejana across equatorial and Easter Microplate boundaries
Source: BMC Evol Biol. 2016 Oct 28;16:235. doi: 10.1186/s12862-016-0807-9 (PMC5084463; doi:10.1186/s12862-016-0807-9)
Supplement: Additional file 5: Table S6. — Tajima’s D and Fu’s F S of each locus in each sample. Table S7: Tajima's D and Fu’s F S of each genetic locus across three geographical groups. Table S8. Isolation with Migration 2-population analyses. Estimated effects of Equatorial and Easter Microplate boundaries on demographic parameters. Maximum likelihood estimates (MLE) of six demographic quantities. Figure S2. Posterior probability estimates for population demographic quantities of the model parameters of IMa2 2-population analyses. Posterior probability curves of divergence time (t) (A), migration rates (2Nm) (B), and effective population sizes (N) (C) between NEPR and SEPR are illustrated on the upper panel, and same posterior curves for the quantities between SEPR and PAR are shown in (D), (E), and (F). The geographical groups of N in figures (C) and (F) are represented as subscripts: n, NEPR; s, SEPR; p, PAR; and a, ancestral population. (DOCX 309 kb) [file 12862_2016_807_MOESM5_ESM.docx]

**Additional file 5**

**Table S6.** Tajima's *D* and Fu’s *F*s of each locus in each sample.

|  |  | 23N | 21N | 13N | 9N | 11S | 14S | 17S | 18S | 32S | 38S |
| --- | --- | --- | --- | --- | --- | --- | --- | --- | --- | --- | --- |
| *mtCOI* | *D* | 0.05 | 0.43 | -1.19 | -0.58 | -1.61 | 0.00 | -1.09 | -1.71 | 0.22 | 1.17 |
|  | *F*_S_ | -0.46 | -0.16 | 0.13 | -4.36 | -2.81 | 0.20 | -3.58 | -1.41 | -0.05 | 0.87 |
| *SAHH* | *D* | -1.09 | -1.13 | -0.69 | -1.74 | -0.72 | -1.13 | 1.64 | -1.46 | -1.07 | 0.79 |
|  | *F*_S_ | -0.18 | -1.60 | -0.59 | -2.38 | -0.41 | 0.95 | 2.34 | -0.04 | -0.21 | 1.31 |
| *GlobX* | *D* | -1.42 | -1.80 | -1.31 | -1.52 | 0.79 | 0.17 | -0.94 | 1.17 | 2.48 | -0.36 |
|  | *F*_S_ | -0.13 | -0.60 | -1.00 | -0.97 | 1.31 | -0.13 | -0.74 | 1.20 | 3.38 | 0.50 |
| *PGM* | *D* | -0.82 | -0.54 | -1.04 | -0.31 | 0.00 | 0.34 | -1.56 | 0.94 | -0.42 | 0.04 |
|  | *F*_S_ | 0.20 | 0.99 | 0.44 | 3.47 | 0.00 | 0.38 | 1.22 | 2.34 | 1.14 | 1.60 |
| *AP_NC1* | *D* | 1.57 | 0.40 | 1.17 | 0.53 | -0.53 | -0.93 | -1.11 | -1.16 | -0.59 | 0.00 |
|  | *F*_S_ | 1.89 | 0.44 | 0.41 | -0.57 | -0.01 | 0.00 | -0.34 | -0.96 | -0.10 | 0.00 |
| *AP_NC3* | *D* | -0.09 | -0.15 | -0.97 | -1.06 | 1.08 | 1.39 | 0.20 | 1.26 | 1.49 | 0.12 |
|  | *F*_S_ | -0.51 | -0.56 | -2.40 | -3.17 | -0.10 | 0.02 | -0.84 | 0.95 | 0.65 | 0.25 |
| *AP_NC8* | *D* | 0.16 | 0.32 | 0.01 | 0.00 | -0.45 | 0.00 | -1.11 | -1.16 | 0.00 | 0.00 |
|  | *F*_S_ | 0.55 | 0.64 | 0.42 | 0.00 | 0.08 | 0.00 | -0.34 | -0.79 | 0.00 | 0.00 |
| *AP_NC20* | *D* | 0.00 | -0.40 | 0.01 | -1.20 | 0.02 | -1.13 | 1.17 | -0.84 | -0.77 | -0.45 |
|  | *F*_S_ | -0.01 | -0.78 | 0.42 | -2.81 | 0.46 | -0.86 | 0.87 | -0.82 | -0.72 | 0.08 |
| *AP_NC22* | *D* | 0.79 | -0.78 | 0.44 | 0.77 | 0.02 | 1.63 | 0.00 | 1.47 | 0.05 | -0.28 |
|  | *F*_S_ | 0.05 | -2.35 | 0.08 | 0.54 | -0.86 | 0.54 | 0.00 | 1.47 | -0.39 | -0.71 |
| *AP_NC28* | *D* | -0.47 | -0.12 | 0.08 | 1.35 | 0.86 | 1.91 | 1.23 | 1.45 | 1.47 | 1.22 |
|  | *F*_S_ | 0.43 | 0.69 | -0.11 | 1.66 | 1.43 | 2.76 | 1.19 | 1.97 | 1.91 | 1.58 |
| *AP_NC32* | *D* | 1.31 | -1.12 | 0.58 | -0.13 | -0.59 | 0.34 | 1.52 | 2.90 | **-2.26** | -1.16 |
|  | *F*_S_ | 1.25 | -2.09 | 0.72 | -1.50 | 1.51 | 0.38 | 5.72 | 9.68 | 4.14 | -0.70 |
| *AP_NC43* | *D* | -1.71 | -1.16 | -1.56 | -1.71 | 0.00 | 0.00 | -1.05 | -0.17 | 0.00 | 0.00 |
|  | *F*_S_ | **-2.60** | -0.79 | -1.96 | -2.60 | 0.00 | 0.00 | -0.18 | 0.30 | 0.00 | 0.00 |

The bold cases of Tajima’s *D* and Fu’s *F*_S_ values are s statistically significant at *α* = 0.004 and *α* = 0.001, respectively; with Bonferroni correction.

**Table S7.** Tajima's *D* and Fu’s *F*s of each genetic locus across three geographical groups.

|  |  | NEPR | SEPR | PAR |
| --- | --- | --- | --- | --- |
| *mtCOI* | *D* | -0.63 | **-2.32** | 0.22 |
|  | *F*_S_ | -5.07 | **-11.57** | 0.16 |
| *SAHH* | *D* | -1.09 | -0.19 | -0.31 |
|  | *F*_S_ | -2.62 | 0.36 | 0.73 |
| *GlobX* | *D* | -1.42 | 1.10 | 1.31 |
|  | *F*_S_ | -2.04 | 1.74 | 1.27 |
| *PGM* | *D* | -0.77 | -0.22 | -0.36 |
|  | *F*_S_ | 0.59 | 0.77 | 1.67 |
| *AP_NC1* | *D* | 0.77 | -1.05 | -0.81 |
|  | *F*_S_ | 0.24 | -1.58 | -0.60 |
| *AP_NC3* | *D* | -0.75 | 0.24 | 0.77 |
|  | *F*_S_ | -2.47 | -0.38 | 0.21 |
| *AP_NC8* | *D* | 0.23 | -1.16 | 0.00 |
|  | *F*_S_ | 0.73 | -1.81 | 0.00 |
| *AP_NC20* | *D* | -1.32 | -0.75 | -0.69 |
|  | *F*_S_ | -4.11 | -1.22 | -0.73 |
| *AP_NC22* | *D* | -1.18 | -0.15 | 0.30 |
|  | *F*_S_ | -5.02 | -0.59 | 0.07 |
| *AP_NC28* | *D* | 0.49 | 2.10 | 1.67 |
|  | *F*_S_ | 1.18 | 3.20 | 2.49 |
| *AP_NC32* | *D* | -1.12 | 1.44 | **-2.46** |
|  | *F*_S_ | -3.67 | 5.59 | 1.34 |
| *AP_NC43* | *D* | **-2.00** | -0.48 | 0.00 |
|  | *F*_S_ | **-8.47** | -0.15 | 0.00 |

The bold cases of Tajima’s *D* and Fu’s *F*_S_ values are statistically significant at *α* = 0.004 and *α* = 0.001, respectively; with Bonferroni correction.

**Table S8.** Isolation with Migration 2-population analyses. Estimated effects of Equatorial and Easter Microplate boundaries on demographic parameters. Maximum likelihood estimates (MLE) of six demographic quantities

|  | Equatorial (NEPR vs. SEPR) | | |  | Easter Microplate (SEPR vs. PAR) | | |
| --- | --- | --- | --- | --- | --- | --- | --- |
|  | MLE | L^1^ | U |  | MLE | L | U |
| N_n_^2^ | 1.75 | 1.15 | 2.65 | N_s_ | 0.36 | 0.20 | 0.60 |
| N_s_ | 0.60 | 0.35 | 0.93 | N_p_ | 0.19 | 0.08 | 0.37 |
| N_a_ | 1.06 | 0.59 | 1.73 | N_a_ | 1.11 | 0.28 | 7.09 |
| 2N_n_m_n_^3^ | 1.29* | 0.23 | 2.88 | 2N_s_m_s_^5^ | 0.82*** | 0.36 | 1.59 |
| 2N_s_m_s_^4^ | 0.19 | 0.00 | 0.88 | 2N_p_m_p_^6^ | 0.45*** | 0.18 | 0.89 |
| t (My) ^7^ | 0.59 | 0.26 | 1.89 | t (My) | 3.30 | 2.61 | 28.82 |

^1^ Lower (L) and upper (U) bounds for 95% highest posterior density (HPD).

^2^ Effective population size of NEPR as a unit of one million individuals. The subscript ‘n’ stands for the geographic group, NEPR. Likewise, the subscripts, s, p, and a, stand for the other geographic groups, SEPR, PAR, and ancestral population, respectively.

^3^ m_n_, migration rate into NEPR from SEPR.

^4^ m_s_, migration rate into SEPR from NEPR.

^5^ m_s_, migration rate into SEPR from PAR.

^6^ m_p_, migration rate into PAR from SEPR.

^7^ Splitting time in million years.

**P* < 0.05, ***P* < 0.01, and ****P* < 0.001

**
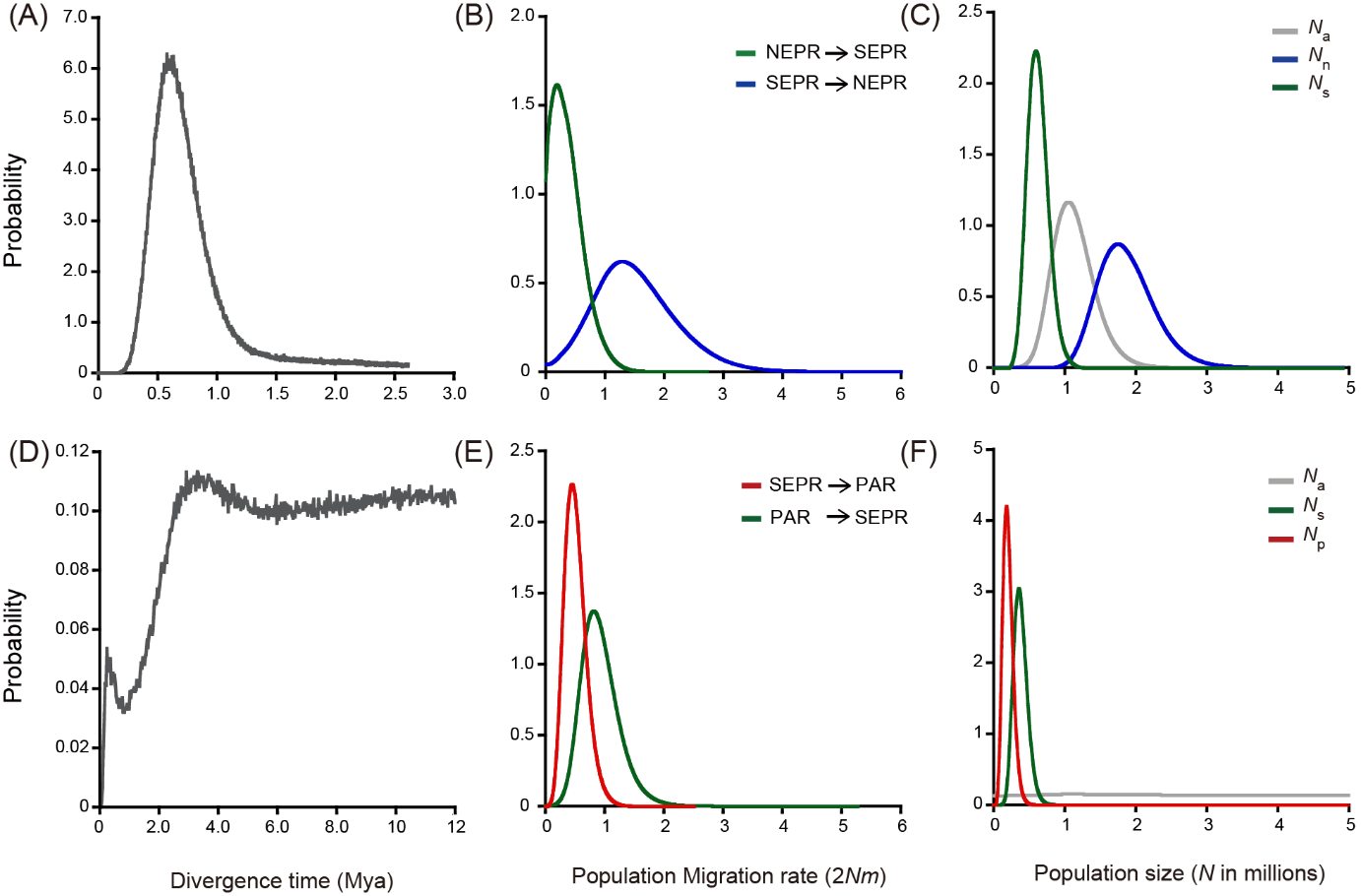
**

**Figure S2. Posterior probability estimates for population demographic quantities of the model parameters of IMa2 2-population analyses.** Posterior probability curves of divergence time (*t*) (A), migration rates (2*Nm*) (B), and effective population sizes (*N*) (C) between NEPR and SEPR are illustrated on the upper panel, and same posterior curves for the quantities between SEPR and PAR are shown in (D), (E), and (F). The geographical groups of *N* in figures (C) and (F) are represented as subscripts: n, NEPR; s, SEPR; p, PAR; and a, ancestral population.
